# The Effect of Resveratrol on Cell Viability in the Burkitt’s Lymphoma Cell Line Ramos

**DOI:** 10.3390/molecules23010014

**Published:** 2017-12-21

**Authors:** Paola Jara, Johana Spies, Constanza Cárcamo, Yennyfer Arancibia, Gabriela Vargas, Carolina Martin, Mónica Salas, Carola Otth, Angara Zambrano

**Affiliations:** 1 Instituto de Bioquímica y Microbiología, Facultad de Ciencias, Universidad Austral de Chile, Valdivia 5090000, Chile; paolad2864@gmail.com (P.J.); johana.spies@gmail.com (J.S.); constanza.carcamoz@gmail.com (C.C.); yennyfer.arancibia@gmail.com (Y.A.); gabs.vargas@gmail.com (G.V.); monsalas@gmail.com (M.S.); 2 Escuela de Tecnología Medica, Universidad Austral de Chile, Sede Puerto Montt 5480000, Chile; carolina.martin@uach.cl; 3 Instituto de Microbiología Clínica, Facultad de Medicina, Universidad Austral de Chile, Valdivia 5090000, Chile; cotth@uach.cl; 4 Center for Interdisciplinary Studies on the Nervous System (CISNe), Universidad Austral de Chile, Valdivia 5090000, Chile

**Keywords:** resveratrol, cell viability, Ramos cells, DNA damage, DNA repair

## Abstract

Resveratrol is a polyphenolic natural compound produced by a variety of crops. Currently, resveratrol is considered a multi-target anti-cancer agent with pleiotropic activity, including the ability to prevent the proliferation of malignant cells by inhibiting angiogenesis and curtailing invasive and metastatic factors in many cancer models. However, the molecular mechanisms mediating resveratrol-specific effects on lymphoma cells remain unknown. To begin tackling this question, we treated the Burkitt’s lymphoma cell line Ramos with resveratrol and assessed cell survival and gene expression. Our results suggest that resveratrol shows a significant anti-proliferative and pro-apoptotic activity on Ramos cells, inducing the DNA damage response, DNA repairing, and modulating the expression of several genes that regulate the apoptotic process and their proliferative activity.

## 1. Introduction

Resveratrol (3,5,4′-trihydroxystilbebe or RSV) is a polyphenolic natural product generated by a wide variety of crops, including grapes, peanuts, plums and berries, and some derived products such as red wine and fruit juice [1,2,3].

Accumulating evidence shows that RSV consumption may have many beneficial properties to human health, acting as an antioxidant, anti-aging, immunomodulator, anti-inflammatory and cardioprotective agent, reducing the risk of coronary artery disease. It has also been postulated to be a mimetic factor for the effects of caloric restriction on metabolism, including the enhancement of insulin sensitivity [4,5,6,7].

In addition, RSV has prompted a great interest in the biomedical industry mainly due to its anti-carcinogenic activity, whereby it can prevent the proliferation of cancer cells, inhibit angiogenesis and reduce invasive and metastatic factors [8,9,10]. Furthermore, some studies have shown that RSV inhibits tumor initiation, promotion, and progression [11].

The antiproliferative and proapoptotic activity of RSV has been reported in different human cancer cell lines that include colon, prostate, and breast cancer as well as leukemia [12,13,14,15]. Although there is abundant data regarding the chemopreventive role of RSV in many cancers, relatively little information exists on the antiproliferative activity of RSV in human lymphoma cells [16,17,18].

Although RSV is a promising multi-target anticancer agent with pleiotropic activities, its specific mechanisms of action remain unclear [19]. In general, RSV targets a great number of intracellular molecules implicated in apoptosis induction. For example, RSV induces cell death by altering proteins of the Bcl-2 family. Specifically, it is able to upregulate the expression of pro-apoptotic proteins such as Bak, Bax, NOXA and PUMA, while lowering the expression of anti-apoptotic members such as Bcl-2, Mcl-1 and Bcl-XL [20,21,22]. Moreover, RSV increases cellular apoptosis by modulating the extrinsic pathway that relies on binding of ligands such as FASL or TRAIL [6,23,24], whereas it also activates the intrinsic apoptotic pathway by inducing the mitochondrial release of cytochrome C, the generation of ROS, and the modulation of the p53 pathway [20,25,26,27].

Interestingly, RSV binds and activates the serine protein kinase ataxia telangiectasia mutated (ATM), inducing autophosphorylation and substrate phosphorylation [28]. ATM activates signaling checkpoints upon genotoxic stress, especially DNA double strand breaks. Indeed, the loss of ATM activity has been observed in various tumor types. RSV is able to induce extensive DNA damage and more specifically DNA double strand breaks in human colon carcinoma cells [29,30]. Also, RSV works on blocking topoisomerase (TOPO) activity. Indeed, RSV induced a delay in S-phase progression with the concomitant phosphorylation of the histone H2AX (H2A histone family, member X) [29,31]. Specifically, in B-cell lymphocytes, DNA double-strand breaks (DSBs) are generated in all developing lymphocytes, a process that is essential for normal lymphocyte development [32]. DSB activates an ATM-dependent signaling pathway that leads to phosphorylation and inactivation of the transcriptional coactivator CRTC2 [33]. CRTC2, in turn, regulates many target genes, some of which are involved in processes that modulate GC B-cell proliferation, self-renewal and also inhibit plasma cell differentiation [34].

In the case of lymphomas, Jazirehi et al., reported that RSV downregulates two anti-apoptotic proteins, Bcl-XL and Mcl-1, whereas it upregulates the pro-apoptotic proteins Bax and Apaf-1 in the Burkitt’s lymphoma cell line Ramos [35]. RSV also promotes cell growth inhibition in NALM-6 cells [36] and blunts PI-3K signaling and glucose metabolism in germinal center-like LY1 and LY18 human diffuse large B-cell lymphomas (DLBCLs) [17]. However, the molecular mechanisms of action of RSV in lymphoma cells remain largely unknown.

In this study, we analyzed the effect of RSV on cell viability in the Burkitt’s lymphoma cell line Ramos. We demonstrate that RSV has anti-proliferative and pro-apoptotic effects on these cells. We show that RSV induces the DNA damage response; activation of DNA repair; and modulates the expression of genes with key roles regulating the apoptotic process and the proliferative activity in this model cell line.

## 2. Results

### 2.1. RSV Induces Decrease in Cell Viability in Ramos Cells

Even though many beneficial properties of RSV have been reported, including anti-proliferative and pro-apoptotic activity in different human cancer cell lines, its effects in human lymphoma cells are poorly described.

**Figure 1:**
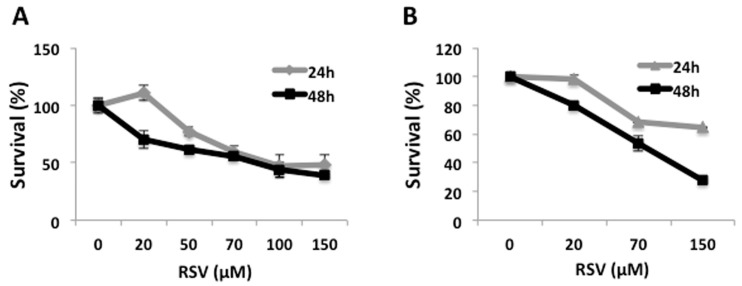
Effect of Resveratrol on cell viability in Ramos cells. Ramos cells were treated with resveratrol (RSV) at the indicated concentrations. (**A**) Cell viability was evaluated by MTT assay during 24 h and 48 h of treatment with resveratrol; (**B**) Cell viability was evaluated with the Trypan Blue exclusion assay during 24 h and 48 h of treatment with resveratrol. Data are presented as mean ± SD, for three independent experiments. * *p* < 0.05, ** *p* < 0.01, compared to control cells.

To determine whether RSV has an antiproliferative effect on Ramos cells, we incubated this cell line with different concentrations of RSV for 24 h and 48 h, and determined viability using the MTT assay. Cell viability decreased in a concentration-dependent manner in response to RSV, by 25% with 50 µM RSV up to 60% using 150 μM RSV at 24 h, and by 30% with 20 μM RSV up to 60% using 150 μM RSV at 48 h (Figure 1A). Similar results were obtained independently using the Trypan Blue exclusion assay to determine cell survival, showing a maximum antiproliferative activity with 150 μM RSV at 24 h and 48 h of treatment (Figure 1B). These data demonstrate that RSV effectively reduces cell proliferation and viability in Ramos cells. For subsequent experiments we chose concentrations between 50 μM and 100 μM, which decrease cell viability by 50% after 24 h of treatment.

### 2.2. RSV Induce Apoptotic Cell Death in Ramos Cells

**Figure 2:**
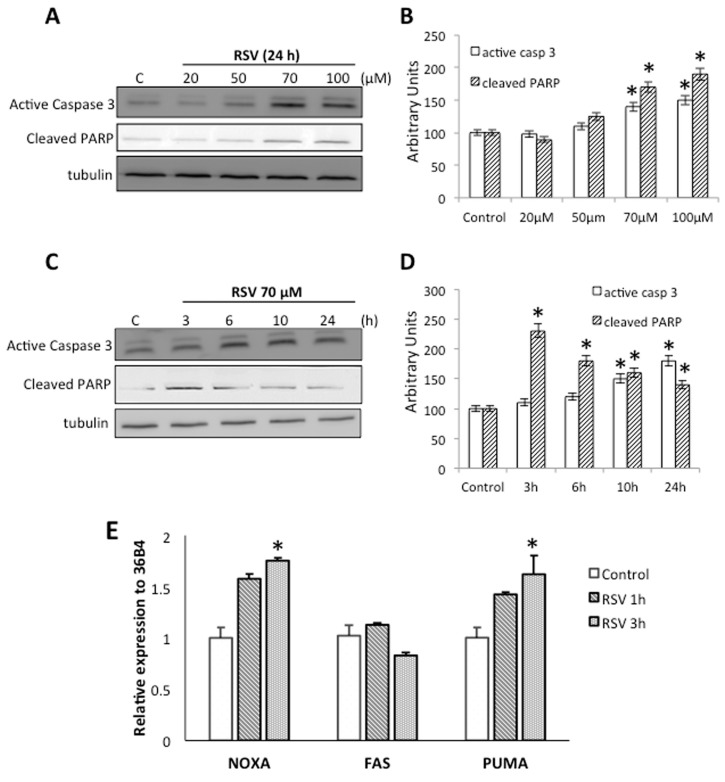
Effect of Resveratrol on apoptosis markers. (**A**) Western blot analysis of proteins from cells treated with different concentrations of resveratrol (RSV) for 24 h using anti-active caspase 3 and anti-cleaved PARP (poly ADP-ribose polymerase) antibodies. Antibodies against total tubulin were used as loading control. (**B**) Quantification of the proteins shown in panel A is represented as a column plot with error bars. (**C**) Western blot analysis of proteins from cells with 70 μM RSV for different time periods using anti-active caspase3 and anti-cleaved PARP antibodies. Antibodies against total tubulin were used as loading control. (**D**) Quantification of the proteins shown in panel C is represented as a column plot with error bars. (**E**) The mRNA levels of apoptotic-related genes were measured by quantitative reverse transcriptase polymerase chain reaction (NOXA; Phorbol-12-myristate-13-acetate-induced protein 1, FAS; Tumor necrosis factor receptor superfamily member 6 and PUMA; p53 up-regulated modulator of apoptosis). Cells were treated with 70 μM RSV for 1 h or 3 h. Results are representative of three independent experiments, * *p* < 0.05 compared to control cells.

RSV exhibits many different mechanisms of action and apoptosis-related targets in various models. To determine whether RSV induces the apoptotic process in Ramos cells, we performed an immunoblotting assay against some important apoptotic markers, specifically, active-caspase 3 and fragmented PARP proteins after different RSV treatment. Antibodies against tubulin were used as loading control. Indeed, RSV triggered a significant increase in active-caspase 3 and cleaved-PARP using 70 μM and 100 μM for 24 h (Figure 2A,B). Treating Ramos cells with 70 μM RSV for different time periods also revealed a significant increase in active-caspase 3 and cleaved-PARP over 3h treatment (Figure 2C,D).

Interestingly, it has been reported that RSV is able to upregulate the expression of several pro-apoptotic mediators [21,22] therefore we treated Ramos cells with 70 μM RSV for 1 h and 3 h, and determined the mRNA levels for NOXA (Phorbol-12-myristate-13-acetate-induced protein 1), Fas (Tumor necrosis factor receptor superfamily member 6), and PUMA (p53 up-regulated modulator of apoptosis) by means of RT-qPCR. Our results shown that RSV induce a significant increase in the mRNA levels of NOXA and PUMA, but has no effect on the expression of Fas (Figure 2E). These results indicate that RSV can activate caspase 3 inducing the fragmentation of its downstream target, and upregulate the expression of a subset of genes know to be linked to apoptotic events.

### 2.3. RSV Induces DNA Damage and DNA Repair in Ramos Cells

**Figure 3:**
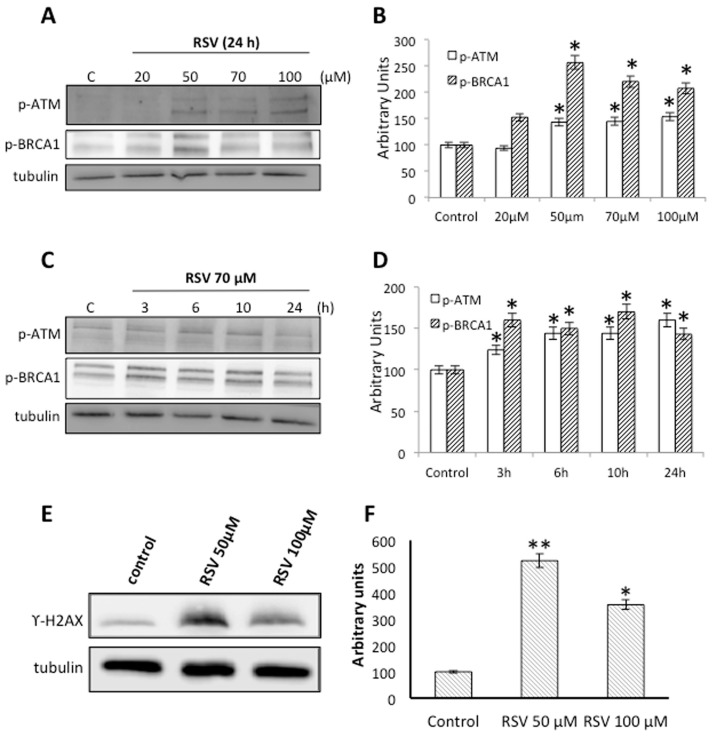
Effect of Resveratrol on the activation of the DNA damage response. (**A**) Western blot analysis of proteins from cells treated with different concentrations of resveratrol (RSV) for 24 h using anti-p-ATM (ataxia-telangiectasia mutated kinase) and anti-p-BRCA1 (breast cancer type 1 susceptibility protein) antibodies. Antibodies against total tubulin were used as loading control. (**B**) Quantification of the proteins shown in panel A is represented as a column plot with error bars. (**C**) Western blot analysis of proteins from cells treated with 70 μM RSV for different time periods using anti-p-ATM and anti-p-BRCA1 antibodies. Antibodies against total tubulin were used as loading control. (**D**) Quantification of the proteins shown in panel C is represented as a column plot with error bars. (**E**) Western blot analysis of proteins from cells treated with 50 μM or 100 μM RSV for 24 h using anti-γ-H2AX antibodies. Antibodies against total tubulin were used as loading control. (**F**) Quantification of the proteins shown in panel E is represented as a column plot with error bars. Results are representative of three independent experiments, * *p* < 0.05 compared to control cells.

Many reports suggest that RSV can induce extensive DNA damage, specifically DNA double strand breaks (DSB), in some tumor cells lines [29,30]. To determine whether RSV is able to induce DNA damage in Ramos cells, we treated cells with different concentrations of RSV for 24 h and tested the phosphorylation levels of ATM (ataxia-telangiectasia mutated kinase) and BRCA1 (breast cancer type 1 susceptibility protein), two proteins associated with the activation of the DNA damage response. Our results show that treatment with RSV over 50 μM induces a significant increase in p-ATM and p-BRCA1 (Figure 3A,B). Also, treating Ramos cells with 70 μM RSV for different time periods revealed a significant increase in ATM and BRCA1 phosphorylation over 3 h treatment (Figure 3C,D). We also treated Ramos cells with two different concentrations of RSV (50 μM and 100 μM) to detect the presence of γ-H2AX. Our results show that treatment with RSV induces a significant increase in γ-H2AX using both concentrations (Figure 3E,F). These data suggest that the induction of DNA damage might be one of the molecular mechanisms involved in the loss of cell viability caused by RSV in Ramos cells.

**Figure 4:**
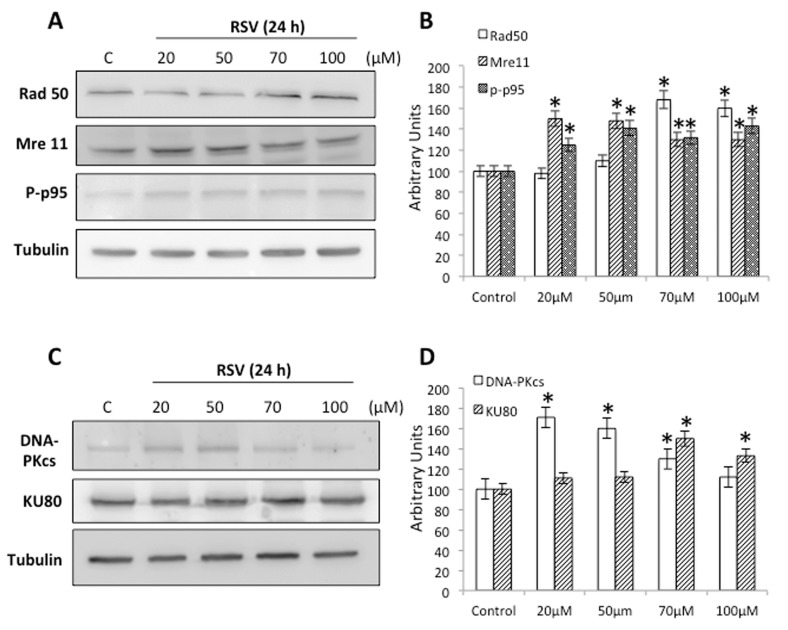
Effect of Resveratrol on the activation of double-strand break repair pathways. (**A**) Western blot analysis of proteins from cells treated with different concentrations of RSV for 24 h using anti-Rad50, anti-Mre11 and anti-p-p95/NBS1 antibodies. Antibodies against total tubulin were used as loading control. (**B**) Quantification of the proteins shown in panel A is represented as a column plot with error bars. (**C**) Western blot analysis of proteins from cells treated with different concentrations of RSV for 24 h using anti-DNA-PKcs and anti-KU80 antibodies. Antibodies against total tubulin were used as loading control. (**D**) Quantification of the proteins shown in panel C is represented as a column plot with error bars. Results are representative of three independent experiments, * *p* < 0.05 compared to control cells.

Non-homologous end-joining (NHEJ) and homology-directed repair (HDR) are the main pathways in all organisms for repairing DBS [37,38]. To determine whether RSV is able to induce the repair mechanisms in Ramos cells, we treated cells with different concentrations of RSV for 24 h and tested the protein levels of Rad50, Mre11 and p-p95/NBS1, proteins that represent the primary DSB sensor. Our results show that treatment with RSV induces a significant increase in all these proteins, suggesting that RSV induces their expression (Figure 3A,B). We also treated cells with different concentrations of RSV for 24 h and tested the protein levels of DNA-PKcs and KU80, essential proteins in the initiation of NHEJ repair pathway. Our results show that treatment with RSV induces a significant increase in both proteins, suggesting that RSV induces the activation of this pathway (Figure 4).

### 2.4. RSV Regulates Gene Expression Related to Proliferation and B Cell Differentiation

DNA double-strand breaks (DSB) are generated in all developing lymphocytes, and it is an essential event for normal lymphocyte development [32]. This process is led by the inactivation of the transcriptional coactivator CRTC2 [33,34], which controls many direct target genes, some of them involved in processes that regulate GC B-cell proliferation, self-renewal, and inhibit plasma cell differentiation.

**Figure 5:**
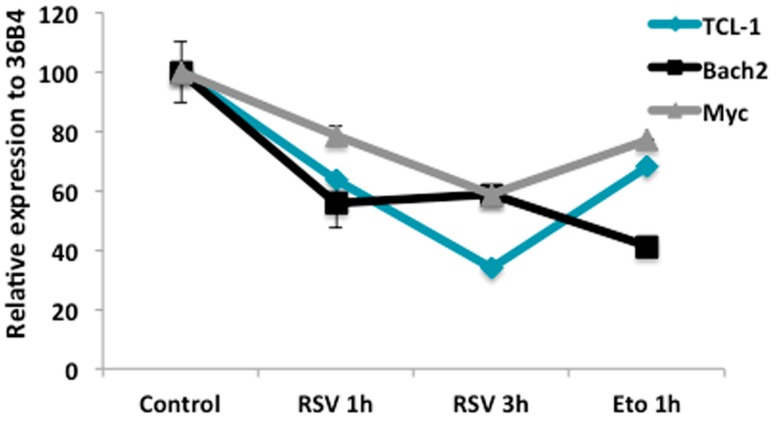
Effect of Resveratrol on the expression of proliferative genes essential in lymphomagenesis. The mRNA levels of proliferative were measured by quantitative reverse transcriptase polymerase chain reaction. Cells were treated with 100 μM resveratrol (RSV) for 1 h or 3 h. The graph represents the relative expression of TCL-1 (T-cell leukemia/lymphoma protein 1A), Bach2 and Myc genes. Results are representative of three independent experiments, * *p* < 0.05.

To determine whether RSV regulates B cell differentiation by reducing the expression of genes involved in B-cell proliferation, we measured three transcripts that are widely studied as reporters of this process: TCL-1 (T-cell leukemia/lymphoma protein 1A), Bach2 and Myc. We treated cells with 50 μM or 100 μM RSV for 24 h and then analyzed mRNA expression using RT-qPCR. We observed a significant decrease in the mRNA levels of TCL-1, Myc and Bach2 (Figure 5). Analogous results were obtained with Etoposide (Eto), a positive control for DNA damage that is capable of downregulating CRTC2-target genes [33].

## 3. Discussion

Resveratrol is a multi-target anti-cancer agent with pleiotropic activity. In this study, we assessed the effects of RSV on cell death in the Burkitt’s lymphoma cell line Ramos. We show that, as in other model cell lines, RSV has anti-proliferative and pro-apoptotic activities in these cells. Furthermore, our results demonstrate that RSV induces the DNA damage response, DNA repairing and modulates the expression of genes with key roles in the apoptotic process and the proliferative activity of Ramos cells.

Resveratrol is a polyphenolic natural product generated by a wide variety of plants. It has prompted great interest in the biomedical community mainly due to its anti-carcinogenic properties. Resveratrol prevents the proliferation of cancer cells by inducing inhibition of tumor initiation, promotion, and progression [10,11]. Although there is accumulating evidence on the chemopreventive role of RSV in many cancers, further data in relation to human lymphoma cells is still lacking [12,13,14,15,16,39].

Here we show that RSV reduces proliferation and cell viability in Ramos cells (Figure 1), concomitant with the induction of caspase-3 and PARP fragmentation (Figure 2), which are normally associate with apoptotic cell death. These results are consistent with previous studies demonstrating that RSV promotes cell growth inhibition in NALM-6 cells [36]; induces cytotoxicity in human Burkitt’s lymphoma, Raji, and Daudi cell lines [40]; and also induces cell-cycle arrest in germinal center-like LY1 and LY18 human diffuse large B-cell lymphomas (DLBCLs) [15,17]. Also, in mantle cell lymphomas (MCL), specifically in jeko-1 cell line, resveratrol induces apoptosis modulating several key molecules involve in cell cycle and apoptosis [41].

RSV exhibits different mechanisms of action implicated in cell cycle control and apoptosis induction. For example, it interacts directly with the human GLUT1 hexose transporter thus inhibiting the transport of hexoses [42]. In addition, RSV treatment results in decreased glycolytic flux, with a parallel reduction in the expression of several mRNAs encoding rate-limiting glycolytic enzymes [39]. Also, RSV induces cell death by altering the expression Bcl-2 family proteins [20], upregulating the expression of Bax, Bak, PUMA, and NOXA, whereas decreasing the anti-apoptotic members Bcl-2, Mcl-1, and Bcl-XL [21,22]. We observed similar results in Ramos cells, where RSV upregulated the expression of genes associated with apoptotic events (PUMA and NOXA). We also analyzed the expression of Fas receptor, where, in contrast to previous studies in ALCL cell lines, where RSV increased cellular apoptosis by enhancing Fas/CD95 expression in a dose-dependent manner [23,24], we did not find major changes in the expression of Fas receptors in Ramos cells.

Another relevant molecular pathway induced by RSV is DNA damage. Our results show a robust activation of the DNA damage response, with increased phosphorylation of the damage sensor ATM (Figure 3A,B), increased p-BRCA1 (Figure 3C,D), and the concomitant phosphorylation of γ-H2AX (Figure 3E,F). ATM activates cell cycle checkpoint signaling upon genotoxic stress, especially DNA double strand breaks. Interestingly, loss of ATM activity is a hallmark of various tumor types and RSV can bind to ATM increasing autophosphorylation and substrate phosphorylation [28,43]. BRCA1 is an essential tumor suppressor involved in DSB repair, preserving genome stability. Ours results are consistent with published evidence suggesting that RSV induces extensive DNA damage, more specifically, DNA double strand breaks in human colon carcinoma cells [29,30]. Interestingly, a well-documented anticancer mechanism of RSV is through the inhibition of topoisomerase (TOPO) activity. Indeed, RSV induced a delay in S-phase progression with the concomitant phosphorylation of histone H2AX [29,31], which is fully consistent with our results. A principal effect of the DNA damage response (DDR) is to maintain genomic stability inducing DNA repair. NHEJ and HDR seem to be the major pathways trigged by DDR in eukaryotic cells. The MRN complex formed by MRE11/RAD50/NBS1 proteins is essential for DNA end resection during HDR repair [38]; our results show a clear increased in the levels of these proteins induced by RSV (Figure 4A,B). At the same time, our results show a clear increased in the levels of DNA-PKcs and KU80 induced by RSV, proteins involved in the initiation of NHEJ pathway [44]. These results demonstrate that DDR induced by RSV in able to promote both NHEJ and HDR repair pathway to maintain genome stability. Importantly, to use different drugs that generate DSBs is extremely beneficial in cancer chemotherapy, increasing the impact of RSV as a complementary therapy.

DNA double-strand breaks are essential for the normal development and differentiation of lymphocytes [32], which is also apparent in Ramos cells as a critical step to induce plasma cell differentiation [33,34]. It has been demonstrated that DNA damage regulates signaling pathways that lower the expression of many genes associated with proliferation and survival. For example, TCL-1 [45], Bach2 [46], and Myc [47] are all downregulated during differentiation in these cells. Our results show decreased mRNA levels for TCL-1, Myc, and Bach2, comparable with the effect of Etoposide, a TOPO2 inhibitor that induce DSBs in Ramos cells [33].

In summary, the results presented here suggest that RSV is a natural molecule with a significant anti-proliferative and apoptotic activity on Ramos cells, inducing the DNA damage response and modulating the expression of several genes that regulate the apoptotic process and the proliferative activity in this model lymphoma cell line.

## 4. Materials and Methods

### 4.1. Cell Culture

Ramos cells, a B lymphocyte cell line from Burkitt’s Lymphoma (ATCC CRL-1596), was grown in RPMI-1640 (Hyclone, Logan, UT, USA) containing 10% fetal bovine serum (FBS, Hyclone, Logan, UT, USA), 50 U/mL penicillin, 50 mg/mL streptomycin, and 2 mM L-glutamine, 1mM Sodium Pyruvate, and essential amino acids (from Hyclone, Logan, UT, USA), at 37 °C in humidified 5% CO_2_ atmosphere.

### 4.2. Cell Viability Assays

Ramos cells were seeded in 96-well plates and treated with different concentrations of resveratrol (Sigma Chemical, St. Louis, MO, USA), we used DMSO as vehicle. After 24 h incubation, mitochondrial activity was measured by the modified 3-[4,5-dimethylthiazol 2-yl]-2,5 diphenyltetrazolium bromide (MTT) assay [48]. Cells were incubated for 4 h at 37 °C with MTT (10 μL of 5 mg/mL MTT solution per well, Sigma-Aldrich, St. Louis, MO, USA). The reaction was stopped with the addition of cell lysis buffer (50% dimethylformamide and 20% SDS, pH 7.4). ΔA values at 550–650 nm were determined using an automatic microtiter plate reader (Metertech Σ960) and the results were expressed as a percentage of control. Cell viability was also assayed by Trypan Blue exclusion and the results were expressed as a percentage of control. The RSV concentrations used in this work are in accordance with previous reports [42].

### 4.3. Western Blot Analysis

Cells were cultured and treated with resveratrol for different time periods and different concentrations. Next, cells were lysed in RIPA buffer (50 mM Tris, pH 7.5, 150 mM NaCl, 5 mM EDTA, 1% NP-40, 0.5% sodium deoxycholate, 0.1% SDS, 100 mg/mL PMSF, 2 mg/mL aprotinin, 2 mM leupeptin, and 1 mg/mL pepstatin) and protein concentration was determined using the Bradford assay. Protein extracts were resolved by SDS–PAGE (60 mg per lane) on a 10% polyacrylamide gel and transferred into immobilon membranes (Millipore, Bedford, MA, USA). After blocking with 5% skimmed milk, membranes were incubated with 1:1000 dilutions of primary antibodies. We used antibodies against p-ATM, p-BRCA1 and γ-H2AX (Cell Signaling Technology, Inc., Danvers, MA, USA) as markers of DNA damage response; active-caspase 3 and cleaved PARP (Cell Signaling Technology, Inc., Danvers, MA, USA) to determine apoptosis marker; and finally, Rad50, Mre11, p-p95/NBS1, DNA-PKcs, and KU80 (Cell Signaling Technology, Inc, Danvers, MA, USA) as markers of DNA repair; Tubulin (Calbiochem, Darmstadt, Germany) was used as loading control.

### 4.4. Quantitative Real-Time Reverse Transcriptase-Polymerase Chain Reaction (RT-qPCR)

Total RNA was isolated from cells using Trizol reagent (Life Technologies, Waltham, MA, USA) following the manufacturer’s instructions. Total RNA was subjected to RT-PCR. 1–5 μg of total RNA was used to synthetize first-strand cDNAs with the iScript kit (BIO-RAD, CA, USA). Quantitative RT-PCR (RT-qPCR) analysis was performed as described previously [49]. Expression was normalized to a 36B4 mRNA control sequence. Oligonucleotide primers for real-time RT-PCR are: TCL-1 FW 5′-CGATACCGATCCTCAGACTCCAGTT-3′, RV 5′-AAAGGAGACAGGTGCTGCCAAG-3′, Myc FW 5′-AGCGACTCTGAGGAGGAACAAGAAGAT-3′, RV 5′-TTGGCAGCAGGATAGTCCTTCCG-3′, Bach2 FW 5′-CTTGCCTGAGGAGGTCACAGC-3′, RV 5′-AGCATCCTTCCGGCACACAAA-3′, 36B4 FW 5′-TGGCAGCATCTACAACCCTGAAGT-3′, RV 5′-TGGGTAGCCAATCTGAAGACAGACA-3′.

### 4.5. Statistical Analysis

Data are presented as mean ± SE of the values from the number of experiments performed in triplicate as indicated in the corresponding figures. Data were analyzed for statistically significant differences (*p* < 0.05) using the Student’s t-test.
